# NF-κB Duplications in the Promoter-Variant HIV-1C LTR Impact Inflammation Without Altering Viral Replication in the Context of Simian Human Immunodeficiency Viruses and Opioid-Exposure

**DOI:** 10.3389/fimmu.2020.00095

**Published:** 2020-01-31

**Authors:** Rajnish S. Dave, Haider Ali, Susmita Sil, Lindsey A. Knight, Kabita Pandey, Lepakshe S. V. Madduri, Fang Qiu, Udaykumar Ranga, Shilpa Buch, Siddappa N. Byrareddy

**Affiliations:** ^1^Department of Pharmacology and Experimental Neuroscience, University of Nebraska Medical Center, Omaha, NE, United States; ^2^Molecular Biology and Genetics Unit, Jawaharlal Nehru Center for Advanced Scientific Research, Bangalore, India; ^3^Department of Biostatistics, College of Public Health, University of Nebraska Medical Center, Omaha, NE, United States

**Keywords:** HIV-1C, SHIV, opioids, heroin, NF-κB, LTR

## Abstract

Recent spread of the promoter variant (4-κB) Human immunodeficiency virus-1 clade C (HIV-1C) strain is attributed to duplication of the Nuclear Factor Kappa B (NF-κB) binding sites and potential increased heroin consumption in India. To study the underlying biology of 4-κB HIV-1C in rhesus macaques, we engineered a promoter-chimera variant (4NF-κB) Simian Human Immunodeficiency Virus (SHIV) by substituting the HIV-1C Long Terminal Repeat (LTR) region consisting of 4 NF-κB and 3 Sp-1 sites with the corresponding segment in the LTR of SHIV AD8EO. The wild-type (3NF-κB) promoter-chimera SHIV was generated by inactivating the 5′ proximal NF-κB binding site in SHIV 4NF-κB. CD8-depleted rhesus macaque PBMCs (RM-PBMCs) were infected with the promoter-chimera and AD8EO SHIVs to determine the effects of opioid-exposure on inflammation, NF-κB activation, neurotoxicity in neuronal cells and viral replication. Morphine-exposure of RM-PBMCs infected with SHIVs 4NF-κB, 3NF-κB, and AD8EO altered cellular transcript levels of monocyte chemoattractant protein 1, interleukin 6, interleukin 1β, and Tumor Necrosis Factor α. Of note, divergent alteration of the cytokine transcript levels was observed with these promoter-chimera wild-type and variant SHIVs. NF-κB activation was observed during infection of all three SHIVs with morphine-exposure. Finally, we observed that SHIV AD8EO infection and exposure to both morphine and naloxone had the greatest impact on the neurotoxicity. The promoter-chimera SHIV 4NF-κB and SHIV 3NF-κB did not have a similar effect on neurotoxicity as compared to SHIV AD8EO. All SHIVs replicated efficiently at comparable levels in RM-PBMCs and morphine-exposure did not alter viral replication kinetics. Future *in vivo* studies in rhesus macaques will provide greater understanding of 4-κB HIV-1C viral immunopathogenesis and onset of disease in the central nervous system during morphine-exposure.

## Introduction

Human immunodeficiency virus-1 clade C (HIV-1C) accounts for 50% of HIV-1 infections in the world and nearly 95% of infections in India (1). Furthermore, HIV-1C is also predominant in Nepal, parts of China and Southern and Eastern Africa. Polymorphisms in the HIV-1C long terminal repeat (LTR) are considered to be a contributing factor for the domination of clade C viral strains in these geographical regions. The U3 region of the HIV-1C LTR includes multiple transcription factors binding sites (TFBS) for nuclear factor kappa B (NF-κB), specificity factor 1 (Sp1), nuclear factor of activated cells (NF-AT) and several others. These transcription factors regulate HIV-1C transcription. For example, the Sp1 proteins are crucial for regulating basal level gene expression of the virus and the NF-κB proteins for the induced viral gene expression. As such, any alterations in the HIV-1C LTR may potentially intensify or weaken viral infection and thereby influence the relative fitness levels of a viral strain (2).

Polymorphisms in the HIV-1 LTR are universally observed. Previous studies have characterized polymorphisms in the Sp elements in the enhancer region (3–5). In one study, the relative affinity of the Sp elements for the Sp factors, impacted viral replication depending on the cell type in which the virus replicated. For example, weak Sp binding sites were detrimental for viral replication in a T (Jurkat) cell line as compared to a monocytic (U-937) cell line (3). Furthermore, single nucleotide polymorphisms (SNPs) have also been identified within the CCAAT/enhancer binding protein (C/EBP) site I and Sp site III (4). The 3T SNP resulted from a C-to-T change at position 3 in C/EBP site 1, while the 5T SNP resulted from a C-to-T change at position 5 in Sp site III. Both, 3T and 5T SNPs result in low affinity binding sites, yet HIV-1 LTR-driven gene expression remains effective. It is possible that NF-κB transcription factor binding sites potentially compensate for this low-affinity (4). Duplication of the NF-κB binding site is unique to the clade C viruses. There are three NF-κB binding sites in the LTR of the wild-type clade C viruses (3-κB HIV). However, the newly emerging variant strains possess four NF-κB binding sites (4-κB HIV). In India, the prevalence of the newly emerging strains containing the NF-κB binding site duplication has increased from 2% during 2000–2003 to 20–30% in 2010–2011 (6).

Opioid-abuse may also serve as an additional factor contributing to the increasing prevalence of the newly emerging strains, such as the 4-κB HIV-1C. Drug abuse has increased enormously in India over the past few years, especially the consumption of heroin. Opioids exert their effects via the opioid-receptors, which are members of the G protein-coupled receptor (GPCR) family. These proteins have seven transmembrane domains that are associated with G protein subunits. Natural alkaloids, endogenous opioids and synthetic opioids bind to opioid receptors with distinctive affinities. This variation in binding affinity was originally utilized to classify the opioid receptors as; a) μ opioid receptor (MOR), κ opioid receptor (KOR) or δ opioid receptor (DOR). The MOR is of particular interest due to its high affinity for opioids that are clinically-utilized or recreationally-abused (7). However, it is not clear if the concomitant increase in 4-κB HIV-1C infections is associated with enhanced consumption of narcotic drugs in India and possibly in other geographical regions outside India. Given that opioid receptor-mediated signaling can activate NF-κB pathway, the interplay of NF-κB binding site duplication and opioid abuse is of concern with regards to 4-κB HIV-1C disease progression (6, 8).

The interplay between the enhanced number of NF-κB binding sites in the viral promoter and increased opioid abuse might also impact the manifestation of HIV associated neurocognitive disorder (HAND). A large majority of previous studies examining HAND have focused on clade B infections, while a relatively smaller number of studies have focused on clade C infections. In one study, significant cognitive deficits on most tests were observed in HIV-1C infected individuals as compared to the control group (9). Patients were treatment naïve with a median CD4 cell count of 97. Study groups were matched for age and education (9). Studies utilizing other HIV-1 clade C cohorts worldwide suggest that there is a potentially high risk for moderate to severe HAND complications (1). Regardless of the HIV-1 clade infection, antiretrovirals have a beneficial effect in diminishing HAND complications (10).

SHIV infected rhesus macaques (RM) provide, a reliable animal model to investigate the interaction between viral infection and opioid-abuse, particularly for characterizing disease progression and central nervous system (CNS) complications (11–13). Therefore, in this study, we engineered SHIV AD8EO with HIV-1C LTR carrying 4 or 3 NF-κB binding sites. The corresponding promoter-chimera SHIVs (SHIV 4NF-κB and SHIV 3NF-κB) along with SHIV AD8EO were tested for their replication capacity in cell lines and rhesus macaque CD8-depleted PBMCs (RM-PBMCs) to understand in greater detail viral infection, inflammatory response and neurotoxicity of these promoter-chimera SHIVs with and without morphine-exposure.

## Methods

### Generation of NF-κB Promoter-Chimera SHIVs

**Figure 1:**
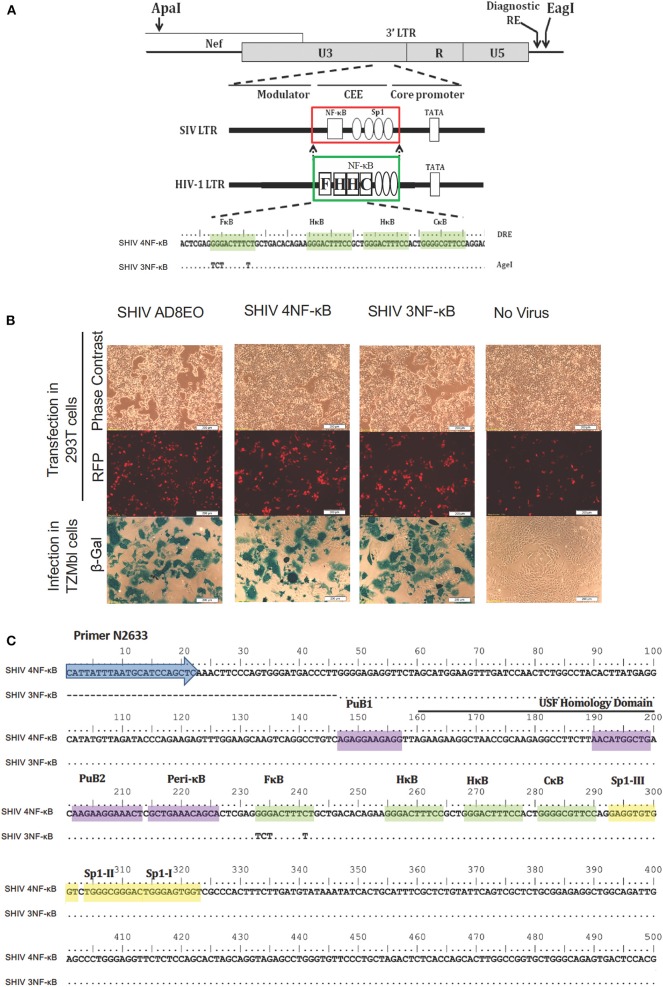
Construction of promoter-chimera SHIVs with NF-κB duplication. **(A)** The schematic diagram of 3′ LTR of SIV AD8EO molecular clone. The locations of *Apa*I and *Eag*I insertion are depicted. The engineered *Apa*I restriction site does not alter the amino acid sequence in Nef. Each variant strain in the panel contains a diagnostic restriction enzyme (DRE) immediately upstream of *Eag*I for unambiguous identification of the clone. The region of SIV comprising of a single NF-κB binding site (square box) and four Sp1 binding sites (ovals) was substituted with an analogous region of HIV-1C LTR comprising of four NF-κB and three Sp1 binding sites (region substituted is highlighted by square boxes). The parental SHIV promoter-chimera LTR containing four NF-κB binding sites (highlighted by shading) was subsequently replaced by variant LTR in which the 5′ most upstream NF-κB binding site was inactivated. In the sequence alignment, dots represent sequence identity. Base substitutions introduced to inactivate the NF-κB binding sites are shown. **(B)** The constructs were transfected into 293T. Transfection efficiency was measured by RFP expressed by EF-1α promoter and infectivity of the progeny virions was determined by LTR driven β-galactosidase expression in TZM-bl cells. **(C)** Sequence confirmation of SHIV AD8EO, SHIV 4NF-κB, and SHIV 3NF-κB molecular clones demonstrates presence of appropriate NF-κB binding sites and other promoter elements.

Using AD8EO, a molecular clone of SHIV (14), SHIV clones with four or three functional NF-κB binding sites (SHIV 4NF-κB and SHIV 3NF-κB) were generated (Figure 1A). The primer pairs N2632 & N2635 and N2633 & N2634 (Table 1) were utilized to synthesize two overlapping PCR products. A unique restriction site, *Eag*I was incorporated in the overlap region (36 bp) of the internal primers N2635 and N2633. Utilizing the external primer pair N2632 and N2634, the overlap PCR product with the *Eag*I restriction site was synthesized. This PCR product was cloned directionally between the *Nsi*I and *Bst*XI enzyme sites.

**Table 1:** Primers for construction of SHIV clones with NF-κB duplication.

**Primer name**	**Primer sequence**
N2632	5'-TATTTAATGCATCCAGCTCAAAC-3'
N2635	5'-CTCTAATGTTGGTCGGCCGTGCTAGGGATTTTCCTG-3'
N2633	5'-GCAGGAAAATCCCTAGCACGGCCGACCAACATTAGAGAAAAG-3'
N2634	5'-TGGGGGCTCCATAAAACTGGTGCTG-3'
N2758	5'-GCCACAGCTATAGCAGTAGCTGAGGGGACAGATAGGG-3'
N2757	5'-GTCTCCCCACGGGCCCGCAAGAGTC-3'
N2756	5'-GACTCTTGCGGGCCCGTGGGGAGAC-3'
N2759	5'-CTTTTCTCTAATGTTGGTCGGCCGTGCTAGGGATTTTCCTG-3'

Next, an *Apa*I site was introduced upstream of the 3′ LTR at position 8849 as per HXB2 utilizing similar overlap PCR strategy. The primer pairs N2758 & N2757 and N2756 & N2759 were utilized to synthesize overlapping 5′-end and 3′-end fragments (Table 1). A unique restriction site, *Apa*I was incorporated in the overlap region (25 bp) of the internal primers N2756 and N2757. Utilizing the external primer pair N2758 and N2759, the overlap PCR product with *Apa*I restriction site was synthesized. This PCR product was cloned directionally between the *BbvC*I and *Eag*I sites engineered above to generate the vector SHIV AD8EOAE (Figure 1B).

Finally, NF-κB variant strains were generated by substituting the original 3′ LTR of AD8EOAE with the variant LTR amplified using a nested-PCR between restriction sites *Apa*I and *Eag*I. Importantly, the original 3′ LTR of AD8EOAE replaced was derived from SIV backbone and contained a single NF-κB binding site followed by four Sp1 binding sites characteristic of the SIV promoter. The chimeric promoters included only the region of the SIV promoter comprising of the single NF-κB and the four Sp1 binding sites and was replaced by the analogous region of the HIV-1C promoter comprising of four NF-κB and three Sp1 binding sites. Thus, the promoter-chimeras contained the upstream modulator region and the downstream sequences preserved from the original SIV background with only the NF-κB-Sp1 region substituted (Figure 1A).

After constructing the FHHC variant containing four NF-κB motifs, the variant with three NF-κB motifs was generated by inactivating the 5′ most NF-κB binding site. Mutations were introduced within the NF-κB binding site sequence to inactivate it while preserving the overall length of the promoter (Figure 1C) (6).

### Viral Stocks

Plasmids encoding NF-κB promoter-variant SHIVs (SHIV 4NF-κB, SHIV 3NF-κB, and SHIV AD8EO) were transfected into HEK293T (ATCC, Manassas, VA) cells with jetPrime™ transfection reagent (Polyplus Transfection, ILLKIRCH, FRANCE). Cell-free viral supernatants were harvested 72 h post transfection. Viral stocks were titered on TZM-bl cells (AIDS Reagent Repository, Germantown MD) and TCID50 values were calculated according to previously described protocol (15, 16).

### Ethics Statement

Adult female Indian-origin RM (Macaca mulatta) were utilized in this study. All animals were maintained at the Department of Comparative Medicine at the University of Nebraska Medical Center in accordance with the rules and regulations of the International Care and Use Committee and according to the guidelines of the Committee on the Care and Use of Laboratory Animal Resources, National Research Council and the Department of Health and Human Services outlined in “Guide for the Care and Use of Laboratory Animals.” All protocols and procedures were performed under approval of the UNMC Institutional Animal Care and Use Committee according to the National Institute of Health guidelines. The animals were fed a monkey diet (Harlan Teklad #2055) supplemented daily with fresh fruits or vegetables and water *ad libitum*. Additional social enrichment, including the delivery of appropriate safe toys, was provided and overseen by the UNMC enrichment staff. Animal health was monitored daily and recorded by the animal care staff and veterinary personnel, available 24 h a day and 7 days a week. Monkeys were caged in and housed in pairs throughout the study. The UNMC primate housing facility has been fully accredited by the Association for Assessment and Accreditation of Laboratory Animal Care International.

### RM-PBMC Isolation and Culture

Naïve animals were anesthetized with an intramuscular injection of 10 mg/kg of ketamine before initiating blood draw. Whole blood was collected from sedated animals. Animals were kept into dorsal recumbency with extended hind limbs. The site for blood collection was the femoral vein in the femoral triangle. Up to 10% of intravascular blood was collected per week and no more than 20% was collected in a month from one animal. Blood was collected in 10 mL BD Vacutainer® EDTA blood collection tubes (Becton Dickinson, Franklin Lakes NJ).

PBMCs were isolated from heparinized whole blood using standard Ficoll-Hypaque centrifugation procedure using Lymphoprep™ (Axis-Shield, Oslo, Norway) (17, 18). RM PBMCs obtained from naïve animals were depleted of CD8+ T cells with CD8 Dynabeads™ (ThermoFisher) according to manufacturer's instructions. CD8 depleted RM-PBMCs were stimulated with 5 μg/mL Concanavalin A (Con A) (Millipore Sigma, St. Louis MO) and 20 U/mL Interleukin 2 (IL-2) (ThermoFisher™, Waltham MA) for 72 h in RPMI media (ThermoFisher) containing 20% heat-inactivated fetal bovine serum (FBS) (Corning, Corning NY), 2 mM Glutamax™ (ThermoFisher) and 50 U/mL of penicillin G, 50 μg/mL streptomycin (P/S) (ThermoFisher). Activated RM-PBMCs were pretreated with 1 μM naloxone (Millipore Sigma) and 0.1 μM morphine (Millipore Sigma) for 4 h. RM-PBMCs infected with SHIV 4NF-κB, SHIV 3NF-κB, and SHIV AD8EO (14) were cultured in IL-2 containing RPMI media. Morphine and naloxone were replenished every 24 h for duration of 20 days.

### Quantitative RT-PCR

Total RNA was isolated from RM PBMCs using Quick-RNA™ MicroPrep kit (Zymoresearch, Irvine CA) according to the manufacturer's instructions. Briefly, 1 μg total RNA was utilized to synthesize complementary DNA as described previously (19). The reverse transcribed RNA was analyzed on the 7500 Fast Real-Time PCR System (Applied Biosystems, Grand Island NY) with the RT2 SYBR Green Fluor qPCR master-mix (Qiagen, Germantown MD). The macaque specific primers were utilized for monocyte chemoattractant protein (MCP-1), interleukin 6 (IL-6), Interleukin 1β (IL-1β) and Tumor necrosis factor-α (TNF-α) and Glyceraldehyde 3-phosphate dehydrogenase (GAPDH) (Table 2). Reactions were performed in triplicates and normalization was done utilizing GAPDH to determine the fold change in expression. The specificity of the qRT-PCR was monitored with a non-template control.

**Table 2:** Primers for qRT-PCR.

**Primer name**	**Primer sequence**
TNF-α forward	5′-CCTCTTCAAGGGCCAAGGCT-3′
TNF-α reverse	5′-GTCTGGTAGGAGACGGCGAT-3′
IL1-β forward	5′-TCAGCACCTCTCAAGCGGAA-3′
IL1-β reverse	5′-AATTGCATGGTGAAGTCAGT-3′
IL-6 forward	5′-GAAGCTGCAGGCACAGAACC-3′
IL-6 reverse	5′-CTGCAGCCACTGGTTCTGT−3′
MCP-1 forward	5′ -AATCAATGCCCCCGTCACTT-3′
MCP-1 reverse	5'-CCCAAGGAAGAACCTCAGGC-3′
GAPDH forward	5′-CAGGCTGGACTGCAGGAACT-3′
GAPDH reverse	5'-ATGACCTTGCCCACAGCCTT-3′

### Cell Fractionation

Cytoplasmic and nuclear fractions were prepared from RM-PBMCs infected with NF-κB promoter-variant SHIVs and control cells, according to previously described protocol (20) with a few modifications. Briefly, cells were washed with PBS, subjected to a hypotonic shock in Buffer A [10 mM HEPES, pH 7.9 containing 0.1 mM EDTA, 0.1 mM EGTA, 10 mM KCl, 1 mM DTT and protease inhibitor cocktail (Sigma Aldrich)]. Subsequently, cells were lysed with 0.5% NP-40 and centrifuged at 6,000 × g for 1 min. This supernatant was utilized as cytoplasmic protein extract. The nuclear pellet was resuspended in buffer D [20 mM HEPES, pH 7.9 containing 0.2 mM EDTA, 150 mM NaCl, 1% v/v glycerol, 1 mM DTT and protease inhibitor cocktail (Sigma Aldrich). Resuspended pellet was passed through QIAshredder™ column (Qiagen) to homogenize. The homogenized lysate was centrifuged at 16,000 × g for 30 min at 4°C to. This supernatant was utilized as the nuclear protein extract.

### Western Blots

Cytoplasmic and nuclear protein extracts obtained from RM-PBMCs infected with NF-κB promoter-variant SHIVs and control cells were subjected to western blot analysis and probed with a polyclonal anti-phospho NF-κB p65 (93H1; Cell Signaling Technology, Danvers MA) or monoclonal GAPDH (1D4, ThermoFisher). Appropriate Goat anti mouse or rabbit secondary antibodies were utilized. Enhanced Chemiluminescence (Azure, Dublin CA) was utilized to detect secondary antibody. Azure c600 western blot imaging system was utilized to capture the chemiluminescent signal. Image Studio™ Lite 4.0 Software (LI-COR, Lincoln NE) was utilized to measure band intensities.

### Neurotoxicity Assay

Retinoic acid differentiated SH-SY-5Y (ATCC) neuroblastoma cells were utilized to generate neurons according to previously published protocol (21). Cells were grown and differentiated in 96 well plates. Each well contained 100 μL of culture medium. Equal volumes (50 μL) of supernatants from RM-PBMCs infected with NF-κB promoter-variant SHIVs were utilized to determine potential neurotoxicity of virions and inflammatory molecules. Neurotoxicity was determined by measuring viability of retinoic acid differentiated neurons with MTT assay (22).

### ELISA

Cell free culture supernatants obtained from RM-PBMCs infected with NF-κB promoter-variant SHIVs and subjected to morphine and naloxone-exposure were utilized. SHIV progeny virion production was monitored by the quantitation of p27 core antigen according to the manufacturer's instructions (XpressBio, Frederick MD).

### Statistical Analysis

Kruskal-Wallis tests (Nonparametric ANOVA) were utilized to determine the statistically significant differences in normalized values of continuous outcomes between treatment groups at each time point separately. Interested two group comparisons were made by Mann-Whitney *U* test if the overall test was significant, adjusting for multiple comparisons with Bonferroni's method. Analysis were performed using SAS version 9.4 (SAS Institute, Cary, NC, United States).

## Results

### Construction of the NF-κB Promoter-Chimera SHIVs

To examine the influence of variation in the copy number of NF-κB binding sites of HIV-1C in the context of the SIV LTR, we engineered the SHIV AD8EO molecular clones and thereby generated the promoter-chimera SHIV 4NF-κB and SHIV 3NF-κB viral strains. Of note, the SHIV and HIV-1C TFBS have structural differences and there are sequence variations within individual TFBS. We previously demonstrated that the central and genetically variant NF-κB binding site (referred to as the C-κB binding site) as well as the Sp1III binding site have co-evolved in HIV-1 and cannot be separated (5). Furthermore, while HIV-1C contains a cluster of three NF-κB binding sites in the enhancer and three Sp1 binding sites in the core promoter, the SHIV clone contains only one NF-κB binding site and four Sp1 binding sites.

Targeted substitutions within the 3′ LTR of SHIV AD8EO were facilitated by engineering two unique restriction sites (*Apa*I and *Eag*I). The resultant molecular clone was designated SHIV AD8E0AE. Next, the SIV LTR encompassing the enhancer region (containing the single NF-κB binding site) and part of the core promoter (containing four Sp1 binding sites) was substituted with the homologous region derived from HIV-1C LTR comprising of four NF-κB and three Sp1 binding sites. Thus, SHIV 4NF-κB was engineered from SHIV AD8EO (Figure 1A). Next, the NF-κB binding site located at the 5′ end of the enhancer (referred to as the F-κB site) in SHIV 4NF-κB molecular clone was inactivated by introducing base substitutions to generate SHIV 3NF-κB (Figure 1A). Integrity of the SHIV LTR upstream of the viral enhancer and downstream of the Sp-1 sites was preserved in SHIV 4NF-κB and SHIV 3NF-κB chimeras. Replication competence of these promoter-chimera SHIVs was further confirmed by transfection in HEK 293T cells. Infectivity of the progeny virions was determined in TZM-bl cells and viral titers were determined. SHIV AD8EO, SHIV 4NF-κB, and SHIV 3NF-κB viruses had similar infectivity as determined by LTR-driven β-galactosidase expression (Figure 1B). In addition, these newly engineered clones were also sequenced to validate the presence of NF-κB binding sites and other promoter elements (Figure 1C).

### Proinflammatory Cytokine and Chemokine Response

**Figure 2:**
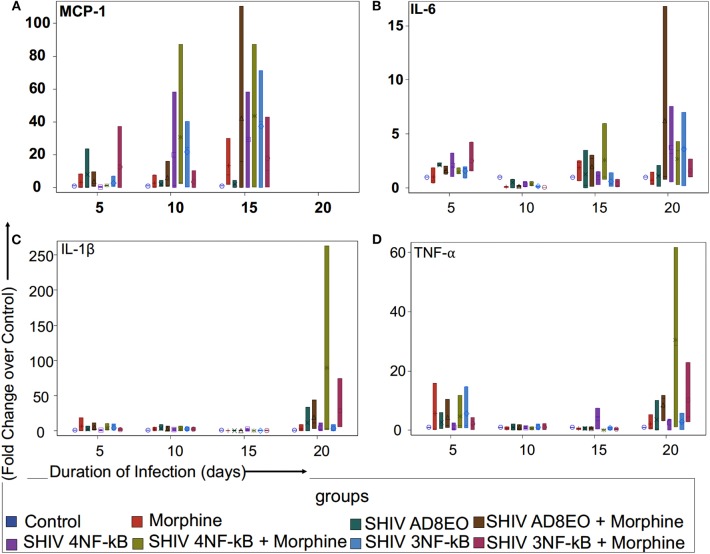
Proinflammatory cytokine and chemokine response during opioid-exposure and SHIV infection of RM-PBMCs. Expression levels of **(A)** MCP-1, **(B)** IL-6, **(C)** IL-1β, and **(D)** TNF-α transcripts was quantitated by qRT-PCR during infection and morphine-exposure of RM-PBMCs with SHIV AD8EO, SHIV 4NF-κB, and SHIV 3NF-κB. The analysis of boxplot showed the distribution of the fold change in expression of each transcript, normalized to GAPDH and relative to the expression of the control group in different treatment groups at each time point. The minimum, first quartile, median, third quartile, and maximum are indicated for each group (*n* = 3).

To determine whether duplications of the NF-κB binding site in the HIV-1C LTR could influence expression of proinflammatory cytokines and chemokine, we utilized RM PBMCs stimulated with ConA/IL-2 for 72 h. These cells were individually infected with equal concentrations of SHIV AD8EO, SHIV 4NF-κB, and SHIV 3NF-κB viral spent media of the 293T cells transfected with the molecular clones. Cellular RNA was extracted from RM-PBMCs infected with one of the three viral strains in the presence or absence of morphine for the qRT-PCR analysis to measure the expression of transcripts of TNF-α, IL1-β, IL-6, and MCP-1. As shown in Figure 2, each of the inflammatory genes exhibited unique response, with MCP-1 transcripts up-regulated early in the infection up to 10 days post infection (dpi) (Figure 2A) and TNF-α transcripts later in the infection at 20 dpi (Figure 2D).

We examined MCP-1 transcript levels in RM-PBMCs infected with each of the three SHIVs separately as well as in uninfected controls (Figure 2A). Analysis with Kruskal-Wallis tests indicated there was no statistically significant difference among eight treatment groups in the median fold change of expression of each transcript at each time point (all *p* > 0.05) (Figure 2A). Based on this test, the *p*-value for the 8-group comparison on 5, 10, and 15 dpi were 0.71, 0.64, and 0.92, respectively. Overall, there was a trend in increase in MCP-1 expression with duration of infection till 15 dpi. At 5 and 10 dpi, SHIV 4NF-κB + Morphine group had elevated MCP-1 expression with a median increase of 1.29-fold (range 1.27–1.37) and 2.91-fold (range 2.34–87.21) as compared to control group, respectively. Furthermore, at 10 dpi even the SHIV 3NF-κB group had elevated MCP-1 expression with a median increase of 24.37 (range 0.32–40.24) as compared to control group. By 15 dpi, all treatment groups had strong upregulation of MCP-1 transcripts. SHIV 4NF-κB and SHIV 3NF-κB treatment groups had higher median increases in MCP-1 expression 29.33-fold (range 0.24–58.42) and 40.24-fold (range 0.16–71.31), while SHIV AD8EO group had a median increase of 1.66-fold (range 0.03–4.5) as compared to control, respectively. Interestingly, while morphine influenced upregulation of MCP-1 transcript for the SHIV 4NF-κB group from median increase of 29.33 to 43.77-fold (range 0.32–87.21), it led to a median decrease in the corresponding SHIV 3NF-κB group of 40.24–10.36-fold (range 0.32–42.97) as compared to control, respectively (Figure 2A).

Next, we sought to assess the expression levels of IL-6 transcript in SHIV infected RM-PBMCs in the presence of morphine. Analysis with Kruskal-Wallis tests indicated there was no statistically significant difference among eight treatment groups in the median fold change of expression of IL-6 at each time point (*p* = 0.11 at day 5, *p* = 0.059 at day 10, *p* = 0.56 at day 15, and *p* = 0.79 at day 20, respectively). Further analysis by Mann-Whitney *U* tests without multiple-comparison adjustment revealed that there was a trend that control group had higher median fold change in IL-6 expression than any other group (all *p* = 0.06) (Figure 2B). As depicted in Figure 2B, there was a trend for overall increase in IL-6 transcript levels as the duration of infection increased. At 20 dpi, the median increase in IL-6 transcript for SHIV AD8EO, SHIV 4NF-κB, and SHIV 3NF-κB groups was 1.19-fold (range 0.17–2.07), 3.2-fold (range 0.55–7.54), and 3.68-fold (range 0.23–6.98) as compared to control, respectively. Interestingly, while morphine influenced upregulation of IL-6 transcript for the SHIV 4NF-κB group from median increase of 3.2–3.44-fold (range 0.33–4.33), it led to a median decrease in the corresponding SHIV 3NF-κB group of 3.68–1.08-fold (range 1.02–2.67) as compared to control, respectively (Figure 2B).

We also determined changes in the IL1β transcript levels during SHIV infection of RM-PBMCs in the presence of morphine (Figure 2C). Analysis with Kruskal-Wallis tests indicated there was no statistically significant difference among eight treatment groups in the median fold change of expression of each transcript at each time point (all *p* > 0.05) (Figure 2C). Based on this test, the *p*-value for the 8-group comparison on 5, 10, 15, and 20 dpi were 0.71, 0.91, 0.11, and 0.32, respectively. As depicted in Figure 2C, there was low level expression of IL1β throughout the infection and there was a surge at 20 dpi. At 20 dpi, the median change in IL1β transcript for SHIV AD8EO, SHIV 4NF-κB and SHIV 3NF-κB groups was 0.02-fold (range 0.02–33.76), 1.93-fold (range 0.03–10.99) and 0.62-fold (range 0.16–8.83) as compared to control, respectively. Morphine led to upregulation of IL1β transcript in all three groups. At 20 dpi, the median increase in IL1β transcript in presence of morphine for SHIV AD8EO, SHIV 4NF-κB, and SHIV 3NF-κB groups was 11.57-fold (range 3.29–43.34), 3.1-fold (range 1.65–262.69), and 5.69-fold (range 5.56–74.62) as compared to control, respectively (Figure 2C).

Finally, we determined changes in TNF-α transcript levels in RM-PBMCs during SHIV infection (Figure 2D). Analysis with Kruskal-Wallis tests indicated there was no statistically significant difference among eight treatment groups in the median fold change of expression of each transcript at each time point (all *p* > 0.05) (Figure 2D). Based on this test, the *p*-value for the 8-group comparison on 5, 10, 15, and 20 dpi were 0.93, 0.99, 0.21, and 0.26, respectively. As depicted in Figure 2C, there was low level expression of TNF-α throughout the infection and there was a surge at 20 dpi. At 20 dpi, the median change in TNF-α transcript for SHIV AD8EO, SHIV 4NF-κB, and SHIV 3NF-κB groups was 0.37-fold (range 0.07–10.08), 3.65-fold (range 0.08–3.71), and 2.59-fold (range 0.18–5.76) as compared to control, respectively. Morphine led to upregulation of TNF-α transcript in all three groups. At 20 dpi, the median increase in TNF-α transcript in presence of morphine for SHIV AD8EO, SHIV 4NF-κB and SHIV 3NF-κB groups was 10.9-fold (range 3.2–11.76), 28.71-fold (range 1.19–61.75), and 22.92-fold (range 2.93–22.92) as compared to control, respectively (Figure 2D).

### NF-κB Activation

**Figure 3:**
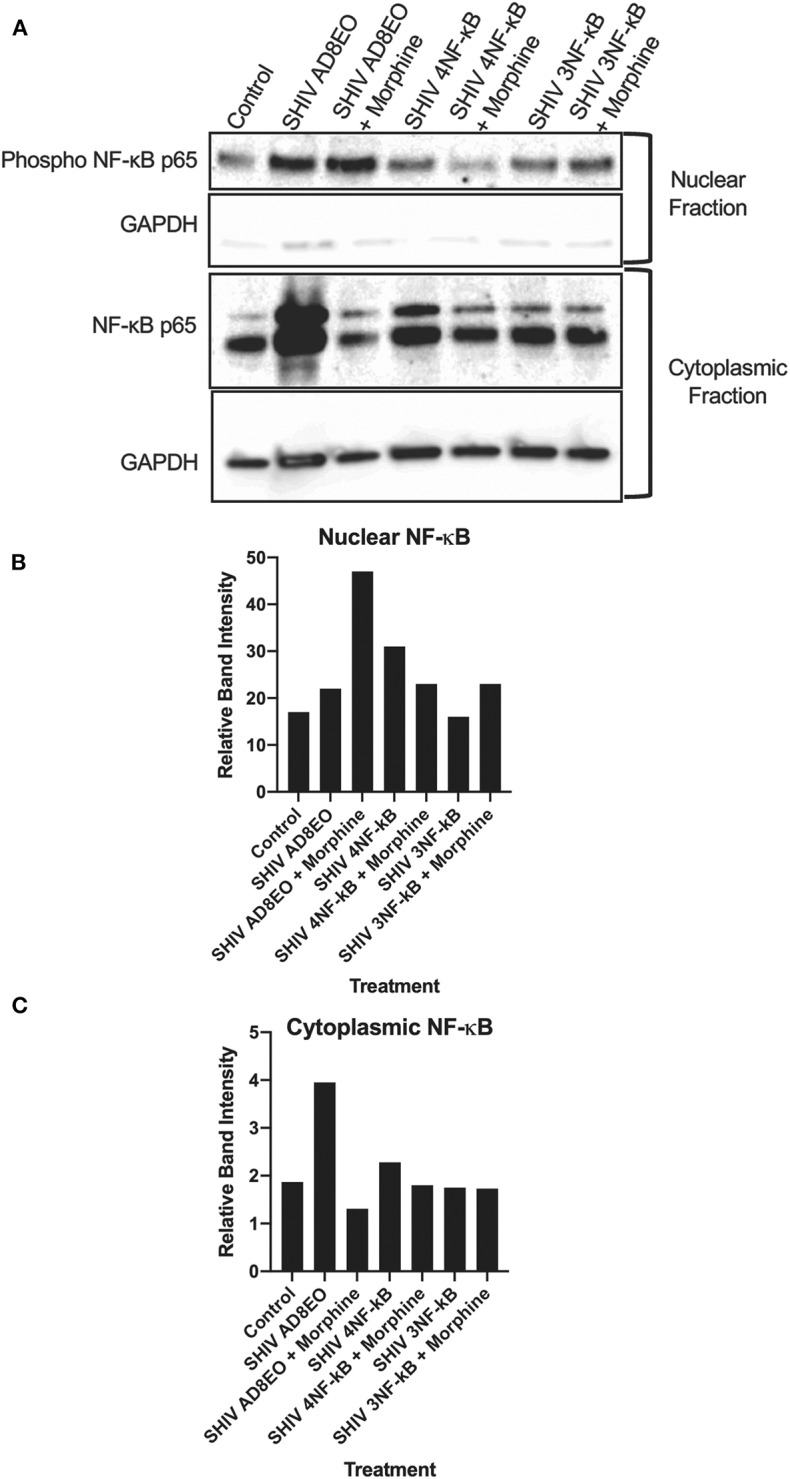
NF-κB activation during morphine-exposure and SHIV infection of RM-PBMCs. **(A)** NF-κB activation during SHIV infection and morphine-exposure of RM-PBMCs with SHIV AD8EO, SHIV 4NF-κB, and SHIV 3NF-κB was determined by detecting translocation of the phosphorylated NF-κB into the nucleus. GAPDH was utilized as a quality control for the extraction of cytoplasmic and nuclear fractions. Relative band intensity was measured with Image Studio Version 4.0 software to determine alterations in NF-κB expression in the **(B)** nuclear and **(C)** cytoplasmic fractions.

Based on the qRT-PCR analyses, we deduced that there was a trend for upregulation of the transcripts involved in the inflammation response. These responses increased with the duration of infection and in presence of morphine. Therefore, we next, determined whether NF-κB was activated in RM-PBMCs infected with SHIV AD8EO, SHIV 4NF-κB, and SHIV 3NF-κB viruses in the presence of morphine. Cytoplasmic and nuclear proteins were fractionated from PBMCs. Phosphorylated NF-κB p65 protein was detected by Western blot in the cytoplasmic and nuclear protein fractions (Figure 3). From the data it was evident that NF-κB was activated and translocated during infection and during morphine-exposure. GAPDH expression was almost similar in all cytoplasmic fraction samples. Almost no GAPDH expression was detected in the nuclear fraction samples (Figure 3A).

In the cytoplasmic fraction, strongest NF-κB expression was detected in SHIV AD8-EO RM-PBMCs and weakest signal was detected in SHIV AD8EO RM-PBMCs during morphine-exposure. Similar albeit weaker response was also noted in SHIV 4NF-κB infected RM-PBMCs and during morphine-exposure. NF-κB band intensities in cytoplasmic fractions of RM-PBMCs from control, SHIV 3NF-κB and SHIV 3NF-κB during morphine-exposure was nearly identical (Figures 3A,C).

Similar to the cytoplasmic fractions, even in the nuclear fractions strongest NF-κB expression was detected in SHIV AD8EO RM-PBMCs. However, morphine-exposure of SHIV AD8EO infected RM-PBMCs led to further increase in translocation of phosphorylated NF-κB into the nuclear fraction. Similar albeit weaker translocation of phosphorylated NF-κB was observed in SHIV 3NF-κB infected RM-PBMCs during morphine-exposure. Interestingly, an opposite response was observed in SHIV 4 NF-κB infected RM-PBMCs during morphine-exposure (Figures 3A,B). Overall based on the analysis of band-intensities, morphine-exposure during SHIV infection led to greatest activation/translocation for AD8EO (36% vs. 17%), 3 NF-κB (23% vs. 19%), and 4 NF-κB (13% vs. 15%). For uninfected control RM-PBMCs, 14% of cytoplasmic NF-κB was activated/translocated to nuclear fraction.

### Neurotoxicity

We inferred that ongoing inflammation and NF-κB activation might also influence viability of neurons. Hence, to further characterize impact of these promoter-chimera SHIVs on neurotoxicity, we performed MTT assays on retinoic acid differentiated SH-SY-5Y neuronal cells using cell-free culture supernatants from RM-PBMC cultures infected with the three SHIVs along with appropriate controls. For these experiments, culture supernatants at 20 dpi were utilized.

**Figure 4:**
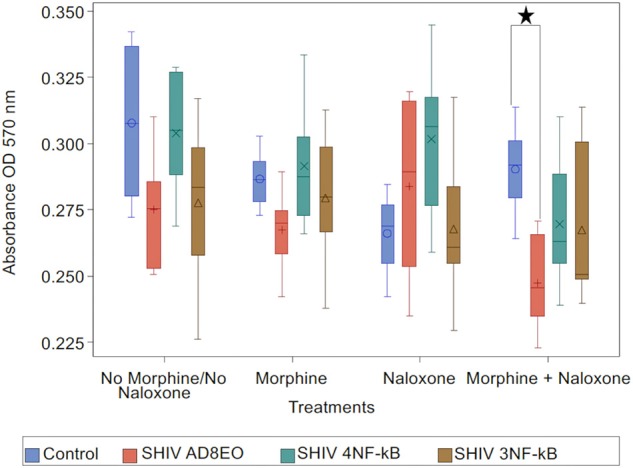
Neurotoxicity of SHIVs generated from RM-PBMCs during opioid-exposure. RA-differentiated SH-SY-5Y neurons were incubated with the cell culture supernatants containing progeny virions generated during SHIV infection and morphine-exposure of RM-PBMCs. Neurotoxicity was assessed by MTT assay. The analysis of boxplot showed the distribution of cell viability in four different virus types under each drug treatment. The minimum, first quartile, median, third quartile, and maximum are indicated for each group (*n* = 6). Statistical significance is indicated as *when *p* < 0.05.

Analysis with Kruskal-Wallis tests indicated there was no statistically significant difference among the four virus groups in the median of cell viability under each drug treatment (*p* = 0.11 for No Morphine/No Naloxone, *p* = 0.19 for Morphine, *p* = 0.24 for Naloxone, and *p* = 0.059 for Morphine + Naloxone, respectively). Further analysis by Mann-Whitney *U* tests without multiple-comparison adjustment revealed that under the treatment of Morphine + Naloxone there was a trend that control group had higher median cell viability than SHIV AD8EO group (*p* = 0.01), SHIV 4NF-κb (*p* = 0.17), and SHIV 3NF-κb (*p* = 0.17); and SHIV AD8EO had lower median cell viability than SHIV 4NF-κb (*p* = 0.13), and SHIV 3NF-κb (*p* = 0.38) (Figure 4).

These results indicate that the combination of SHIV AD8EO infection and exposure to both morphine and naloxone has the greatest impact on the neurotoxicity. The promoter-chimera SHIV 4NF-κB and SHIV 3NF-κB do not have a similar effect on neurotoxicity with the promoter-chimera SHIVs as compared to SHIV AD8EO.

### Replication Kinetics of the NF-κB Promoter-Chimera SHIVs

**Figure 5:**
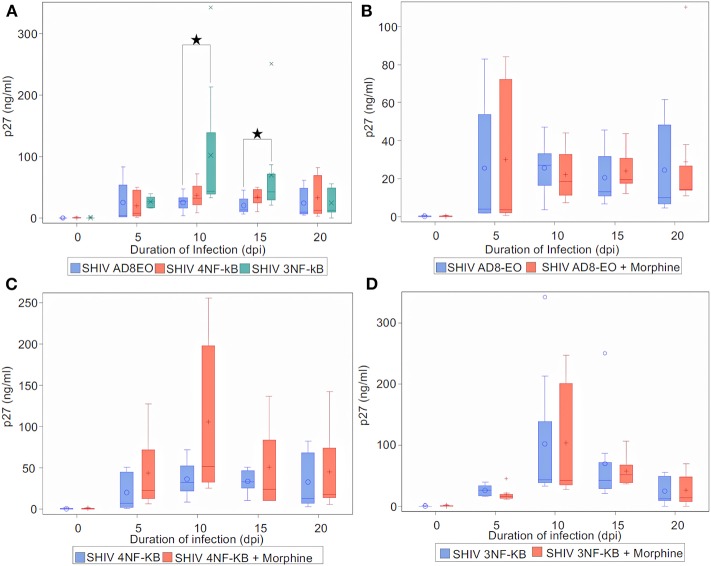
Infectivity of promoter-chimera SHIVs with NF-κB duplication in RM-PBMCs during opioid-exposure. ConA/IL-2 activated and CD8-depleted RM PBMCs were infected with SHIV AD8EO, SHIV 4NF-κB, and SHIV 3NF-κB in the presence of 0.1 μM morphine along with control infection. SIV capsid p27 protein was quantified from the cell culture supernatants using ELISA at the indicated times. **(A)** Replication kinetics of SHIV AD8EO, SHIV 4NF-κB, and SHIV 3NF-κB (*n* = 8). **(B)** Replication of SHIV AD8EO, **(C)** SHIV 4NF-κB, and **(D)** SHIV 3NF-κB during morphine-exposure (*n* = 8). The graph indicates the distribution of p27 levels including the median for each group (*n* = 8). Statistical significance is indicated as *when *p* < 0.05.

Finally, to determine the effect of NF-κB binding site duplication in HIV-1C LTR on viral replication during morphine-exposure, we measured progeny virion production quantitated by p27 ELISA (Figure 5). Cell-free culture supernatants from RM PBMCs infected with equal concentrations of SHIV AD8EO, SHIV 4NF-κB, and SHIV 3NF-κB viruses. In addition, in a set of cells, morphine was also added throughout the infection (Figure 5). Analysis with Kruskal-Wallis tests indicated a statistically significant difference among the three virus groups in the median of p27 at day 10 and day 15 (*p* = 0.03, respectively), but not at day 0, day 5, and day 20. Based on this test, the *p*-value for the 3-group comparison on 0, 5, 10, 15, and 20 dpi were 0.97, 0.57, 0.03, 0.03, and 0.82, respectively. Further analysis by Mann-Whitney *U* tests with Bonferroni correction revealed that the median p27 level in SHIV AD8EO group was lower than SHIV 3NF-κB group at day 10 (Bonferroni-corrected *p* = 0.02), and day 15 (Bonferroni-corrected *p* = 0.05) (Figure 5A).

Next, we sought to determine the effect of morphine-exposure on replication kinetics of these viruses. For each virus type, analysis with indicated there was no statistically significant difference in the median of p27 levels between control and morphine groups at each time point (all *p* > 0.05) (Figures 5B–D). Viral replication was also evaluated during naloxone alone exposure as well as in the presence of both morphine and naloxone For each virus type, analysis with Kruskal-Wallis tests indicated there was no statistically significant difference among the four drug groups in the median of p27 levels at each time point (all *p* > 0.05) (Supplementary Figure 1). Based on these observations, we concluded that morphine-exposure does not impact replication of these viruses.

## Discussion

In this study we report: (1) the engineering of replication-competent promoter-chimera variant (4NF-κB) and wild-type (3NF-κB) SHIV viral strains and (2) morphine-mediated effects on expression of proinflammatory cytokines and chemokines response, (3) neurotoxicity and viral replication utilizing clade C promoter variant SHIVs. Interestingly, divergent alterations in transcript levels of MCP-1, IL-6, and TNF-α were observed for the promoter-chimera SHIVs. The promoter-chimera SHIVs exhibited reduced NF-κB activation as compared to SHIV AD8EO. In addition, we also observed diminished neurotoxicity for the promoter-chimera SHIVs as compared to SHIV AD8EO. Of note, viral replication of the promoter-chimera SHIVs was not found to be influenced by morphine among these SHIVs *in vitro*.

Engineering of SHIV 4NF-κB and SHIV 3NF-κB posed several technical problems. One of the difficulties was the various virus-specific differences in the profile of the TFBS between HIV and SIV. These differences spanned throughout the LTRs including the upstream modulator region, the enhancer, the core promoter and the trans-activation response element. To preserve the basic characteristic of the SIV LTR, we substituted only the enhancer region comprising of the single NF-κB binding site and a part of the core promoter comprising of four Sp1 binding sites with the homologous region derived from the HIV-1C LTR comprising of four NF-κB and three Sp1 binding sites. The 5' most upstream NF-κB binding site was subsequently mutated by introducing inactivating base substitutions. The overall length of the viral promoter in the strains was preserved. The promoter-chimera thus generated consisted of the upstream modulatory region and the regions downstream of the Sp1 sites derived from SIV and the NF-κB-Sp1 region derived from HIV-1C in the backbone of AD8EOAE. Substitution of the entire NF-κB-Sp1 region was necessary given the virus-specific genetic variations in the motifs of the two TFBS families and the obligatory functional association between the central C-κB motif and HIV-1C unique Sp1 III site. Two unique restriction sites (*Apa*I and *Eag*I) were engineered in the AD8EO molecular clone to generate AD8EOAE to permit the targeted substitution of the 3′ LTR. Importantly, the engineering of *Apa*I does not cause an amino acid variation in Nef.

We observed a divergent response in the expression of proinflammatory cytokines and chemokines during infection of RM PBMCs with the promoter-chimera SHIVs in the presence of morphine. Morphine-exposure triggered an increase in MCP-1 transcripts during SHIV 4NF-κB infection and a decrease during SHIV 3NF-κB infection, respectively. Morphine-exposure during SHIV 3NF-κB infection further suppressed IL-6 transcript levels, while during SHIV 4NF-κB infection an increase in IL-6 transcript levels was noted. No such divergence in IL1β transcript levels was observed. Interestingly, while both of the promoter-chimera SHIVs exhibited a suppression in TNF-α transcript levels, this suppression was most potent for SHIV 4NF-κB. The association between duplication of a NF-κB binding site and the divergent expression of proinflammatory cytokines and chemokines is not clear. Therefore, this warrant measuring all the known other proinflammatory cytokines.

NF-κB activation plays a critical role in the survival and differentiation of T-lymphocytes. Dysregulation of the NF-κB pathway has pathological and immunological consequences affecting several diseases and chronic inflammation disorders (23, 24). It was, thus, not surprising to observe the activation of NF-κB during infection by the SHIVs in RM PBMCs. Interestingly, morphine-exposure failed to modulate NF-κB activation significantly in the current study. The absence of NF-κB activation in murine microglia in the presence of morphine (0.1–10 μM) has been reported previously (25). However, in murine microglia, an augmented NF-κB activation in presence of lipopolysaccharides and morphine was observed (0.1–10 μM) (25). The expression of pro-inflammatory cytokines, such as TNF-α and activation of NF-κB are interlinked. Interestingly, similar synergistic activation of NF-κB has been observed in human fetal microglia and astrocytes (26). While we observed upregulation of TNF-α expression, the physiological levels observed in our study are subtle as compared to these *in vitro* experimental investigations. This might perhaps explain why morphine did not exacerbate NF-κB activation to a larger extent.

In this study, we observed diminished neurotoxicity with the clade C equivalent NF-κB engineered promoter chimera SHIVs as compared to clade B SHIV AD8EO that contain one NF-κB site in viral promoter. Differences in progression of HAND have been observed in different clades of HIV, with clade B viruses manifesting a more potent outcome as compared to clade C viruses (27, 28). These differences have not always been consistent (27). Another factor that potentially contributes to the differential progression of HAND in clade C vs. B infections could be attributed to the diminished neurotoxicity of C-tat as compared to B-tat (29, 30). The lower neurotoxicity of the C-tat protein has been attributed to alteration in the dicysteine motif within the neurotoxic region of B-tat (28, 29). Interestingly, the C-Tat of South African Clade C viruses have a higher frequency of the dicysteine motif and higher neurotoxicity (31).

Of note, we did not find enhanced SHIV replication in RM-PBMCs in the presence of morphine-exposure. We utilized an end-step in the viral life cycle (SIV p27 core antigen in cell-free culture supernatants) as a measure of the effect of morphine and naloxone on viral replication. Similar failure of morphine to impact HIV replication has been reported earlier in human monocyte derived macrophages (h-mdms) (18, 32). In contrast, a few other studies have reported augmented replication of HIV R5 isolates in h-mdms (33) and in human neonatal macrophages (34). In the morphine dependent macaque models of SIV infection, rapid CNS disease progression has also been demonstrated (12, 35, 36). Thus, each of these experimental strategies captures different aspect of viral fitness, infectivity or disease progression. Our ongoing studies in rhesus macaques should elucidate in greater detail the interplay of morphine and viral disease pathogenesis for these promoter-chimera SHIVs.

Our study presents the initial characterization of these promoter-chimera SHIVs. Additional studies are necessary to determine the underlying mechanism(s) for the increase in the prevalence of the 4-κB HIV-1C strains. The interplay of opioids and SHIV infection is most pronounced in the expression of proinflammatory cytokines and chemokines. Our ongoing study, in the rhesus macaque model of SHIV infection with morphine dependency will likely be helpful in elucidating the underlying mechanism(s) attributed to increased potency of the 4-κB HIV-1C, disease progression and its effect on the CNS.

To summarize, in this study we generated promoter-chimera SHIVs that include the duplications of NF-κB binding site in the HIV-1C LTR. This duplication is associated with the increased prevalence of 4-κB HIV-1C strain. The promoter-chimera SHIVs were replication competent as assessed in RM-PBMCs. A key finding of this study is the interplay of morphine and SHIV infection resulting in differential expression of selected proinflammatory cytokines and chemokines without affecting viral replication kinetics. Finally, we also noticed diminished neurotoxicity for these promoter-chimera SHIVs as compared to SHIV AD8EO.

## Data Availability Statement

The datasets generated for this study are available on request to the corresponding author.

## Ethics Statement

The animal study was reviewed and approved by the University of Nebraska Medical Center (UNMC) Institutional Animal Care and Use Committee. For details, refer to the Methods section.

## Author Contributions

RD performed RM experiments, analyzed the data, and wrote the manuscript. HA performed cloning experiments. SS performed qRTPCR assay. LK and KP coordinated naïve rhesus macaque bleeds and isolated PBMCs. LM assisted with plasmid and opioid procurement/inventory coordination and FQ performed statistical analyses. UR, SB, and SNB oversaw the study and provided overall direction for the study and edited the manuscript.

### Conflict of Interest

The authors declare that the research was conducted in the absence of any commercial or financial relationships that could be construed as a potential conflict of interest.

## Supplementary Material

Supplementary Figure 1 Replication kinetics of promoter-chimera SHIVs with NF-κB duplication in RM-PBMCs during opioid-exposure. ConA/IL-2 activated CD8-depleted RM PBMCs were infected with SHIV AD8EO, SHIV 4NF-κB, and SHIV 3NF-κB in presence of morphine, naloxone and morphine plus naloxone. SIV capsid p27 was quantified with ELISA in cell culture supernatants at the indicated times. Replication kinetics of **(A)** SHIV AD8EO, **(B)** SHIV 4NF-κB, and **(C)** SHIV 3NF-κB in presence of morphine, naloxone, and morphine plus naloxone along with control infection. The graph indicates the distribution of p27 levels including the median for each group (*n* = 8).
