# Supplementary figures and images for: NF-κB Duplications in the Promoter-Variant HIV-1C LTR Impact Inflammation Without Altering Viral Replication in the Context of Simian Human Immunodeficiency Viruses and Opioid-Exposure
Source: Front Immunol. 2020 Jan 31;11:95. doi: 10.3389/fimmu.2020.00095 (PMC7006833; doi:10.3389/fimmu.2020.00095)

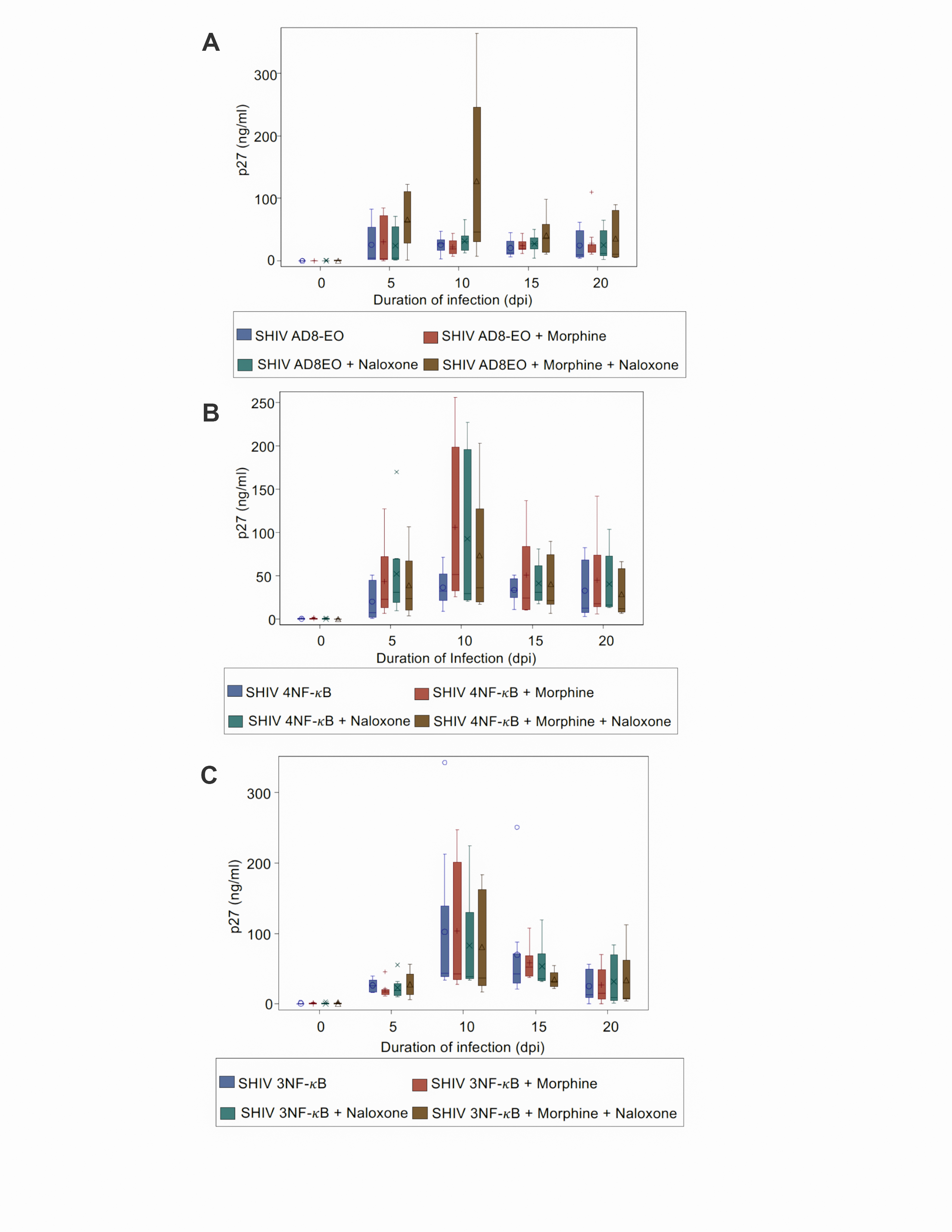

Supplement: Supplementary Figure 1 — Replication kinetics of promoter-chimera SHIVs with NF-κB duplication in RM-PBMCs during opioid-exposure. ConA/IL-2 activated CD8-depleted RM PBMCs were infected with SHIV AD8EO, SHIV 4NF-κB, and SHIV 3NF-κB in presence of morphine, naloxone and morphine plus naloxone. SIV capsid p27 was quantified with ELISA in cell culture supernatants at the indicated times. Replication kinetics of (A) SHIV AD8EO, (B) SHIV 4NF-κB, and (C) SHIV 3NF-κB in presence of morphine, naloxone, and morphine plus naloxone along with control infection. The graph indicates the distribution of p27 levels including the median for each group (n = 8). [file Image_1.TIF]
